# Not Too Old for TikTok: How Older Adults Are Reframing Aging

**DOI:** 10.1093/geront/gnac055

**Published:** 2022-05-05

**Authors:** Reuben Ng, Nicole Indran

**Affiliations:** Lee Kuan Yew School of Public Policy, National University of Singapore, Singapore, Singapore; Lloyd’s Register Foundation Institute for the Public Understanding of Risk, National University of Singapore, Singapore, Singapore; Lee Kuan Yew School of Public Policy, National University of Singapore, Singapore, Singapore

**Keywords:** Aging narratives, Content analysis, Self-portrayals of aging, Social construction of old age, Social media

## Abstract

**Background and Objectives:**

Although reputed for being the virtual playground of teenagers, TikTok has since made its way to older adults, some of whom have become content creators with millions of followers. Despite their immense sway over audiences, as well as their potential to reconfigure socially constructed notions of aging, these older TikTok personalities have been given scant attention in gerontological literature. We fill this gap by exploring how older adults use TikTok to engage in discourses on old age.

**Research Design and Methods:**

We compiled the most viewed videos of users aged 60 and older with at least 100,000 followers, generating 1,382 videos with over 3.5 billion views. Videos that did not feature older adults engaging in discourses on aging were excluded, resulting in 348 videos for content analysis. Both inductive and deductive approaches guided the qualitative analysis.

**Results:**

Three themes emerged: Nearly 3 in 4 videos featured older adults “Defying Age Stereotypes” (71%; Theme 1), 18% featured them “Making Light of Age-Related Vulnerabilities” (Theme 2), and 11% involved older adults “Calling out Ageism” (Theme 3).

**Discussion and Implications:**

This is the first known study exploring how older adults consciously engage in discourses of aging through their participation on TikTok. Our findings highlight the potential for older adults to be put at the vanguard of a movement aimed at challenging socially constructed notions of old age.

Older adults are frequently seen to populate a technophobic group (Jenkins, 2019). However, statistics reveal such beliefs to be blatantly false. An analysis conducted by Pew Research Center found a remarkable increase in the uptake of digital technology by older Americans. Whereas 14 percent of those aged 65 and older were users of the Internet in 2000, 73 percent were in 2019 (Livingston, 2019). Additionally, while only half of adults aged 50 and older owned smartphones in 2014, smartphone adoption today is 86 percent among individuals aged from 50 to 59, 81 percent among those aged from 60 to 69, and 62 percent among those aged 70 and older (Kakulla, 2020).

Available in over 150 countries, TikTok is a microvideo sharing platform famed for its assortment of creative and easy-to-use editing tools (Bresnick, 2019). Since its international debut in 2017, TikTok’s growth has been phenomenal, with the application proceeding to accumulate 507 million global users by the end of 2019 (Bursztynsky, 2021). Usage of TikTok skyrocketed further with the onset of the COVID-19 pandemic, which saw the application edging out its competitors to become the most downloaded application of 2020 (Nakafuji, 2021).

Although TikTok is reputed for being the virtual playground of teenagers (Bresnick, 2019), the latest social media giant has since made its way to the older population (Dellatto, 2019). Some of these older users have become content creators, successfully racking up millions of followers, dispelling the long-held belief that older adults are passive consumers of social media (Leist, 2013). Despite their immense sway over audiences, as well as their potential to reconfigure socially constructed notions of aging, older TikTok personalities have been given virtually no attention in gerontological literature. Our study fills this gap by exploring how older adults use TikTok to engage in discourses surrounding old age.

The theory of social constructionism holds that all human knowledge is created, transmitted, and maintained in social contexts (Berger & Luckmann, 1966). To understand age as a social construction is to understand that while aging itself is a biological process, the meanings imputed to growing old are neither fixed nor immutable (Gergen & Gergen, 2001). Scholars are generally in unison that society prizes youthfulness and views later life as symbolic of vulnerability and decrepitude (Ng et al., 2015). Negative age stereotypes have been found to proliferate in both mainstream (Ng, 2021a; Ng & Chow, 2021; Ng & Tan, 2022; Ng et al., 2021c) and social media (Jimenez-Sotomayor et al., 2020; Levy et al., 2014), reaching an all-time high during the COVID-19 pandemic, during which the virus was labeled a “Boomer Remover” due to the belief that only older people succumbed to it (Skipper & Rose, 2021).

Like other forms of social categorization, the effects of age cannot be understood in isolation. Age interacts with other axes of social differentiation such as gender, class, and race to shape one’s lived experience (Krekula et al., 2018). Regarding gender specifically, older women often find themselves enmeshed in a situation of double jeopardy, whereby the interface between ageism and sexism engenders greater vulnerability (Krekula et al., 2018). This gendered nature of ageism is particularly apparent in the case of outward appearance. While the value of women is frequently measured by whether they are able to maintain some semblance of a youthful look, men’s level of attractiveness tends not to be as age-specific. In fact, aging may at times even enhance men’s overall appeal (Halliwell & Dittmar, 2003).

Subjective aging refers to how older adults make sense of their own aging process (Westerhof et al., 2014). It is well documented that the meanings ascribed to old age have enormous implications on one’s health. According to Levy’s (2009) theory of stereotype embodiment, cognitive and physical health outcomes are influenced by the kinds of age stereotypes internalized by the individual. Negative age stereotypes may reduce one’s sense of self-efficacy, increase the risk of depression and affect both the immune and cardiovascular system. Conversely, positive self-perceptions of aging are associated with improved functional health, well-being, and longevity (Levy, 2009).

Past literature has shown that older people often attempt to distance themselves from the category of old age. For instance, they may eschew the label “old” as a self- protective strategy to dissociate themselves from the stigma of aging (Hurd, 1999). Additionally, they may choose to partake in antiaging interventions like beauty work so as to approximate a youthful image (Clarke & Griffin, 2008). However, there also exists evidence that some older adults actually resist socially constructed notions of aging by looking forward to growing old (Sawchuk, 2009). A recent study found that older people displayed positive views about aging in spite of the prevalence of negative age stereotypes (Malani et al., 2020). Perceiving one’s aging process favorably is generally regarded as a form of cognitive adaptation aimed at maintaining a positive self-concept in a culture where old age is decried (Westerhof & Barrett, 2005).

Presently, inquiry into the use of social media among older adults focuses primarily on its social (Baecker et al., 2014) and cognitive benefits (Quinn, 2018), as well as the motives of older adults for using social media (Sheldon et al., 2021). Other studies have observed how older adults are discursively constructed by the younger demographic on platforms such as Twitter and Facebook (Giest & Ng, 2018; Jimenez-Sotomayor et al., 2020; Levy et al., 2014; Ng & Indran, 2022). While some scholars have studied content creation in later life (Ferreira et al., 2017), research on how members of the older cohort use social media to engage in discourses on old age is still in its inception. To the best of our knowledge, only two studies have delved into this topic. Lazar et al (2017) found that older persons blog to articulate their experiences of ageism as well as to share strategies on how to navigate such experiences. McGrath (2018) uncovered that older women use Instagram and blogs as avenues to achieve greater visibility online as well as to challenge negative age stereotypes.

Conceptually, our study makes an invaluable contribution to the gerontological field, particularly to the emergent area pertaining to how older adults use social media to engage in discourses on later life. With the COVID-19 pandemic having supercharged the usage of TikTok—and more broadly social media—among older persons (Fishwick, 2020), the need to understand how they present themselves on social media is now critical. In terms of practical implications, this study looks at how social media can be harnessed as a venue through which ageism can be combated. Although scholars and policymakers have endeavored to combat ageism through initiatives that involve educating the public or building intergenerational solidarity, few have considered putting older adults front and center in the fight against ageism. By elucidating how they consciously engage in discourses on old age through their participation in social media, we explore the potential for empowering older adults to take on a more active role in ongoing efforts to reframe aging (Ng & Indran, 2021a, 2021d).

Guided by a social constructionist lens, this study asks the following questions: How do older adults engage in discourses surrounding old age on TikTok? In what ways do they use TikTok to negotiate, resist or reproduce socially constructed meanings of old age? To answer these questions, we analyze videos created by older content creators on TikTok.

## Method

### Data Set

Following earlier work (Herrick et al., 2020), a new TikTok account was created to consolidate the videos. This was done to reduce bias since videos on the application are arranged based on a complex algorithm that takes into account the popularity of the video (measured by views, likes, comments, and shares), the popularity of the creator (measured by followers and engagement), any previous content that was engaged with, and the geographical location of the device where the application was accessed. No content was previously engaged with to guarantee a regular user’s experience in using the application (Herrick et al., 2020).

**Figure 1.**
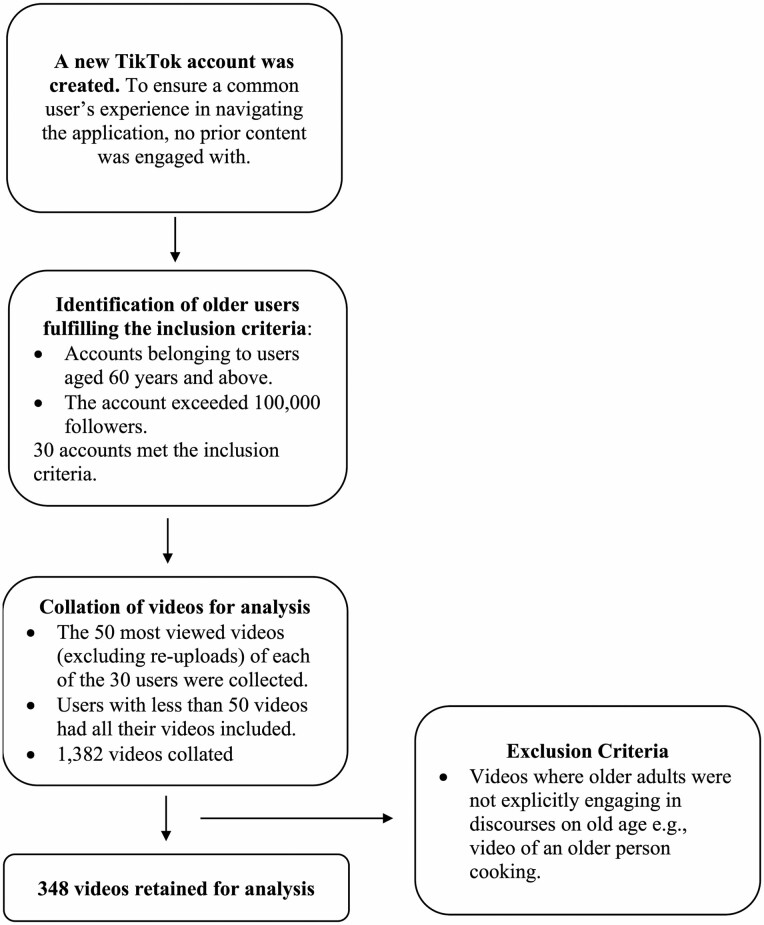
Process of collecting older adults’ videos related to discourses on aging on TikTok.

Videos were crawled with the objective of identifying accounts belonging specifically to older TikTok personalities. These accounts were compiled into a database based on the following inclusion criteria: (a) Account belonged to a content creator aged 60 and older. The age of each content creator was declared in the user’s biography, or in one of the videos he/she had uploaded, or verified through secondary sources such as news articles; (b) The older TikToker’s follower count exceeded 100,000 at the time of analysis. Our original sample comprised 35 accounts, of which 30 met the inclusion criteria. For each account, we collated the 50 most viewed videos excluding reuploads. If the user had fewer than 50 videos, all videos were included. This generated 1,382 videos which received over 3.5 billion views. Videos which did not feature older adults explicitly engaging in discourses on old age were removed. For example, videos where older adults were cooking or sharing about a day in their life were excluded. After applying the aforementioned exclusion criteria, 348 videos were retained for analysis. Each video had a maximum duration of 1 min as mandated by the platform at the time of analysis. Figure 1 provides a flowchart of the video collation process.

### Video Content Coding

Consistent with prior research (e.g., Sipocz et al., 2021), the coding rubric was developed through both inductive and deductive modes of reasoning (Armat et al., 2018). We first identified a set of categories based on existing literature to design a preliminary codebook. The analysis was subsequently conducted in several stages, with each video viewed twice by the authors to ensure familiarity with the data. The aim of the first viewing was to confirm the validity of the initial set of categories, as well as to modify the codebook until all variables were refined and defined clearly. During this first viewing, a new category was added whenever a video featured a particular attribute that could not be suitably coded into any of the existing categories, and appeared frequently enough to warrant its own category. The aim of the second viewing was to make sure we had a framework that was sufficiently representative of the different types of videos in order to finalize the coding rubric.

To ensure rigor in the analysis, the two coders had regular discussions during which any discrepancies were reviewed and adjudicated. Areas of major overlap were identified and sectioned into broader themes. The percentage agreement between the two raters (both authors) was 92.5% with a weighted Cohen’s kappa of 0.91 (*p* < .001), showing high interrater reliability. Three themes emerged from this iterative procedure. Of note, a video may be classified under more than one theme. As mentioned in previous work, categories in a content analysis need not be mutually exclusive although they should be internally homogeneous (i.e., coherent within themes) and externally heterogeneous (i.e., distinct from each other) as far as possible (Bengtsson, 2016).

## Results

### Summary of Insights from Content Analysis of TikTok Videos

**Figure 2.**
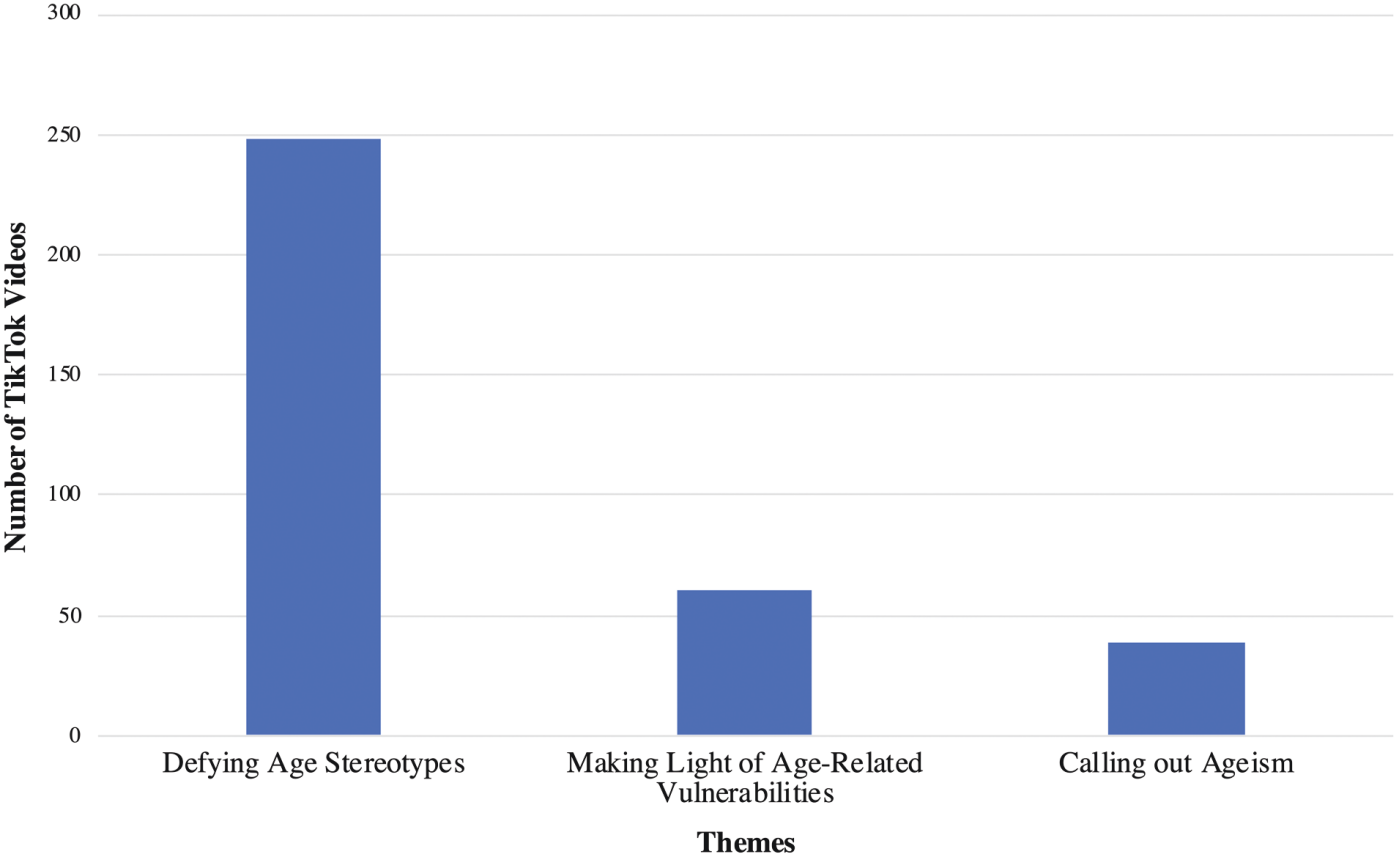
Themes of TikTok videos pertaining to how older adults engage in discourses on old age.

Three themes surfaced from our content analysis of 348 videos where older adults engaged in discourses on old age. Most of the videos (71%; *N* = 248) fell under the theme “Defying Age Stereotypes” (Theme 1). Posts assigned to this theme involved older adults challenging age stereotypes. Examples include protagonists looking forward to growing older or embracing their aging bodies. The theme “Making Light of Age-Related Vulnerabilities” (Theme 2) was present in 18% of the uploads (*N* = 61). Videos of older adults joking about age stereotypes related to dementia, nursing homes, etc., were grouped under this theme. The final theme “Calling Out Ageism” (Theme 3) appeared in 11% of the posts (*N* = 39). Videos revolving around this theme featured older adults explicitly denouncing ageist beliefs or practices. An example of a relevant post would be one of an older person sharing an experience in which he or she was derogated on the basis of age. Figure 2 summarizes the themes while the following paragraphs elaborate on them.

### Defying Age Stereotypes (Theme 1; 71%)

Videos belonging to this theme featured older adults challenging mainstream discourses on old age. One TikToker began her video with a sprightly “I feel cute today! Look at my outfit” before capering about and flaunting her outfit while singing, “I feel good today.” In another clip, the same user filmed herself post haircut, self-assuredly exclaiming, “This is me after my glow up. I look so pretty and I feel so pretty.” At one point of the video, the user even brought the camera closer to her face, making no attempt to camouflage any signs of aging. Responding to the comment “U (*sic*) look beautiful” left by a fellow user in a previous video, a TikToker remarked, “Tell me something I don’t know” before puckering her lips mischievously. This self-confidence was also apparent in a video with the text, “I may be too old or too out of your league,” where a user greeted the audience with a wink over an audio track playing the words, “You (*sic*) liking what you see.” A similar video depicted one user parading along the sidewalk coquettishly in a midriff-baring top, with a caption declaring unabashedly to viewers her age of 70 years.

The “glamma”—a portmanteau combining “glamorous” and “grandma”—was a recurring motif in videos created by two accounts in particular. Videos uploaded by the first account typically depicted an all-women quartet strutting down the streets donned in stylish and at times figure-hugging apparel. Content from the other account involved a self-proclaimed 60-year-old documenting herself decked out in various streetwear, with captions such as “I just hate when people define what a grandma should wear,” “When you are 60, you can be cooler than me,” and “Fashion is not up to age, it’s up to what you like (*sic*).”

Older men too uploaded videos in which they were perfectly happy with their appearance. One such individual created a montage of photos of himself as part of a “self-confidence trend” while another took a video in which he showed off his “cool new look” featuring a bucket hat and a pair of sunglasses. One post was of an older man watching a younger man performing a provocative dance. The former then reciprocated in kind, with the playful caption “I can’t help it if I’m desirable.”

Another set of clips included older adults positioning themselves as superior to their younger counterparts. A case in point is a video captioned “I may be 86 but can still drink more than you lightweights.” The user audaciously performed a series of acts such as chugging alcohol, vaping, and attempting to pole dance. This same user recorded a video of herself recovering from a fall down the stairs with a twerk, a form of dance that involves being in a squat position and thrusting one’s hips. Her caption was “I may be 86 but still twerk better than you.”

There were uploads where individuals celebrated what their aging bodies could accomplish. In a video with the caption “Two knee replacements and still slayed,” a user performed a viral dance routine, preening herself on being unfazed by past bodily ailments. One post portrayed an older man putting his walking stick aside to join his granddaughter in a dance with a text sticker indicating, “I may be 87 but still can’t resist a dance.”

Some users debunked myths pertaining to the physical infirmities of the older cohort. Calling herself a “fitness junkie,” one TikToker had numerous videos of herself taking up a variety of fitness-related challenges on the application, such as the #abchallenge and #cupidshufflechallenge. She also had videos where she filmed herself completing various exercises including weightlifting, planking, and mountain-climbing. Proud of what her body could achieve at the age of 81, the octogenarian made it a point to affix her age as a text sticker to almost all her videos. In a video captioned, “Cut me some slack, I’m 79,” a male TikToker did a bicep curl and in another clip, a user pranced around the backyard spiritedly, remarking in his caption that he had more energy than his granddaughter despite being 87 years of age. One video featured a user dancing with a caption reminding audiences that “it’s important for all ages to keep moving and stay active.”

Certain videos depicted older woman taking ownership of their sexuality. For example, one user described how she would flirt with older men during bingo with a flirtatious, “Are you looking for a wife?” Another TikToker joked about attending church on three consecutive Sundays for the express purpose of flirting with the priest.

There were also posts in which individuals expressed their contentment with their lives. In one such clip, a user performed a dance number with the words, “Me at age 75, living my best life, happy and healthy on social media with 3 million supporters!!” A similar video contained the words, “Me age 87, verified on TikTok, truly happy and thriving with the support of all of you.” In this video, the user acted as his wife looking down at him from heaven, beaming with joy and proud of his accomplishments.

One user talked about not being scared of getting infected by COVID-19 despite recurring depictions in the media of older adults as an at-risk cohort. She insisted on being a “healthy, old (expletive deleted),” while also making it clear that she was not being blasé about the virus as she implied she would adhere to safe distancing measures.

### Making Light of Age-Related Vulnerabilities (Theme 2; 18%)

Videos under this theme featured jocular discussions concerning old age. These discussions circled around topics related to cognitive impairment, physical debilitation, death, the COVID-19 virus, and sexual dysfunction. In a video embedded with a text sticker that read, “My Alzheimers (*sic*) kicking in while scrolling through tik tok (*sic*),” a user dramatized the disorientation experienced by those with Alzheimer’s, specifically through the use of a filter meant to induce a sense of vertigo, as well as her choice of an audio clip in which one song was jarringly layered over another. Similarly, in another video prefaced with a caption that stated, “It’s a joke… we are both healthy,” a user recorded herself brushing her hair “for the sixth time,” chalking up her memory lapses to dementia. The next scene was of her husband completely mesmerized watching his wife doll up, accompanied by the text sticker “Him being amazed because he also has dementia and forgot I have done it 6 times already.” Another video depicted the same TikToker making a prank call to a friend with dementia for the third time in the year to tell her friend to pay her the five hundred dollars still “owed” to her.

One user poked fun at her own susceptibility to falls. With the text “Me facetiming life alert after I have fallen down the stairs and can’t get up,” she filmed herself sprawled lifelessly across the staircase with her spectacles shifted to the side of her face for dramatic effect. A number of TikTokers partook in the “#LeavingMyBody” trend on the application. Set to the upbeat theme song of the American teen sitcom “True Jackson VP,” the trend involves layering the effect of a purple flame over the user to illustrate negative emotions “leaving one’s body” upon receiving a meaningless or dismissive remark. One video portrayed a user skipping down the street with a text sticker announcing, “Old age leaving my body when someone says, ‘you don’t look 87’.” Another video using the same audio–visual template portrayed the TikToker prancing about with the words “the arthritis, hearing loss and tumors leaving my body after being told to walk it off.” Other instances of older persons joking about age-related losses include one where a user pretended to mix up a glass of lemonade with her urine sample, and another in which a user asked God for an age refund.

Some uploads presented older adults putting their own creative spin to viral trends on the application to make light of death. For example, while the “green screen scan” effect—a filter which allows users to superimpose themselves onto other images—is commonly used by individuals to photograph themselves with their younger selves, one TikToker set out to “take a picture with (her) older self.” She scanned herself into a picture of a graveyard to joke about her impending death. This use of morbid humor was further accentuated by her caption which read, “I’m so old (*sic*) follow before I die.” In another clip, a user juxtaposed a scene in which she was dancing along to the lyrics of a song, “I used to be so beautiful (*sic*) now look at me” with another one that saw her lying in a coffin.

The idea that older adults are more susceptible to the COVID-19 infection formed the premise of a handful of videos. A user dramatized her return to “bingo night” after a pandemic-induced hiatus by enumerating the different types of girls who attend bingo night—Karen, a widower, Liz, “the one who eats junk food” and finally, Mary, the one who “didn’t make it through quarantine.” In describing Mary, the user added to the screen a facetious “Rip!” and death-related emojis. In another post, the same TikToker irreverently assumed the part of God handing out “live past 80” cards. This scene then transitioned to another one overlaid with the phrase “God to 90% of my friend group,” timed to coincide with a section of the audio clip with the words “Not for you.”

Sex-related humor was a common denominator in a number of videos. In a 3-part video series, a TikToker listed “erectile dysfunction,” “life alert,” and “foot fungus” as constituting “things that turn boomers on to the max.”

### Calling Out Ageism (Theme 3; 11%)

Many TikTokers narrated their encounters with other users who were unwelcoming of older adults on a platform perceived as exclusive to younger people. For instance, in response to a snide remark, “Go back to Facebook (*sic*) old lady” made by a fellow user on the application, a TikToker sniggered with a voiceover “No.” In another clip, the same user registered her vexation with users who made ageist comments such as “Somebody come get their grandma” or “Boomers don’t belong on tiktok (*sic*).” One individual responded to the comment, “I’ve never seen an 84-year-old on TikTok” with style, embellishing himself with a necklace and a pair of sunglasses—in stark contrast to the rest of his uploads where he was dressed more plainly—splicing this scene with a screenshot of a song titled “What’s Poppin,” a slang which means what is going on.

One user compiled several hate comments leveled at her, some being “Your (*sic*) not ladylike,” “You cant (*sic*) dance” and a constant refrain directed at older users, “Your (*sic*) too old to be on TikTok.” During the first scene of the clip, she held a walker while frowning to appear bothered by the comments. She lost the walker in the subsequent scene, instead choosing to adorn herself with a feather boa and a tiara while confidently retorting, “I am ladylike and a boss (expletive deleted) (expletive deleted).” In this scene, she took a swig of alcohol before blowing a kiss at her detractors.

A user with the biography “Im (*sic*) 89 and probably have more followers than you” acted out a situation where she desperately tried to get past a door that represented TikTok, but with the TikTok community—presumably dominated by younger people—prohibiting her from entering. In this video, she lip-synced to an audio clip pleading, “Let me in” with a tongue-in-cheek “They are just scared of all this” as a caption. Some of these older TikTok personalities found their own contemporaries making ageist comments. One such personality re-enacted an encounter with her friends who judged her decision to post videos on the application as age-inappropriate. “Don’t you think you should act your age?” and “Don’t you think you’re too old to be on TikTok?” were examples of comments she received.

In a video addressed at “the people that (*sic*) make fun of old people,” one TikToker rejoined with an audio clip, “Listen, you’re not a different breed. You’re just different. You’re weird.” The same user expressed his annoyance with individuals who harp on the fact that he is old. Filling up the screen with the quote “You’re old,” the user responded sarcastically with an audio clip of the words “I didn’t even notice” running on a continuous loop.

As a riposte to an offensive comment, “At least I’ll be alive in a few months” left by a user in one of her videos, an individual mouthed the words of a voiceover, “You can’t get rid of me, (expletive deleted), I’m not going nowhere, I’m not going no (expletive deleted) where.” Another video featured an older woman cheekily responding to a hate comment, “Grandmas with tiktok (*sic*) aren’t cute” with an audio template of the words “Promise you I’ll change your mind.” One user shrugged off an ageist remark, “Die already (*sic*) you have lived long enough” saying that although it was unacceptable, his feelings could not be hurt in view of the outpouring of support his followers had extended to him.

Seemingly frustrated at users who repeatedly cautioned her to “use the rails” so that she would not “fall and break something,” one user took a video of herself deliberately avoiding using the rails while rebelliously marching up a flight of stairs to the lyrics “(expletive deleted) being good, I’m a bad (expletive deleted). I’m sick of (expletive deleted) trying to tell me how to live.”

In a clip captioned, “Things that majorly kill my vibe,” a user consolidated a series of ageist comments directed at her. These include “Shut up boomer,” “Don’t you have arthritis?” and “Stick to the nursing home.” The same user called out individuals who referred to her as a grandmother simply because she was in her later years, referencing a larger issue involving the tendency to pigeonhole all older adults as grandparents despite not knowing whether they have grandchildren or even children.

## Discussion

Despite the growing presence of older adults on social media, gerontological efforts to examine how they engage in discourses surrounding later life have yet to gather pace. To narrow this gap in the literature, we sought to articulate the ways in which older adults use TikTok to subvert or perpetuate socially constructed notions of old age. Three themes emerged from our content analysis of videos uploaded by older persons: Defying Age Stereotypes (Theme 1), Making Light of Age-Related Vulnerabilities (Theme 2), and Calling Out Ageism (Theme 3).

Notwithstanding the ubiquity of negative age stereotypes (Ng et al., 2015), unfolding on TikTok is a powerful countercultural phenomenon in which older persons actually contest hegemonic discourses on old age by embracing or even celebrating their aged status. Most of these older TikTokers happen to be women. Despite the reality of gendered ageism (Krekula et al., 2018), these women make no attempt to conceal markers of old age, instead displaying a sense of self-assuredness that belies mainstream constructions of aging as a dreadful process to be delayed or prevented. Additionally, some of these women fiercely resist common stereotypes of older women as passive, mild-mannered, and weak (Grenier & Hanley, 2007), instead opting to present themselves as fierce or even foul-mouthed.

While it is heartening to see some of these older TikTokers defying age stereotypes related to frailty, we acknowledge that such representations of old age may create a slippery slope whereby ableist views are promoted. Scholars have drawn attention to how ageism is reinforced by ableism—the belief that there is a certain physical ideal to which individuals ought to conform (Berridge & Martinson, 2017). Sustained efforts should therefore be made to ensure that current initiatives to reframe aging factor in the heterogeneity of older adults so as to avoid perpetuating the dual stigma of ageism and ableism.

There is considerable evidence that ageist stereotypes preponderate among the young on social media (Jimenez-Sotomayor et al., 2020; Levy et al., 2014). The participation of older adults in social media is therefore vital in ensuring that such ageist ideas are not left unchallenged. Eleven percent of the videos analyzed featured older adults calling out ageism among younger people and even among their own contemporaries. Some of these older people lay claim to their right to use TikTok, resisting the socially constructed notion that using the application is only appropriate for younger individuals. What this indicates is that older adults are actively using social media as a way to destabilize age-related norms, complementing ongoing efforts to combat ageism (Ng & Indran, 2021a, 2021d).

Older adults are constant targets of disparaging and ageist jokes (Palmore, 2003). It is thus intriguing that a select group of older people rely on old age as fodder for comic relief in their TikTok material, albeit on a platform that prides itself on levity and irreverence (Mediakix, 2019). Humor has been conceptualized as a multidimensional construct with both adaptive and maladaptive elements (Demjén, 2016). While some have found that older adults may take offense at jokes which depend on age-related susceptibilities as a punchline (Greengross, 2013), others conclude that older adults may take jabs at their own vulnerabilities to gain mastery over an otherwise distressing situation (Clarke & Wolverson, 2016). Even as the use of such humor may entrench ageist notions of later life, it may also serve as an attempt by older adults to negotiate the stigma of old age, thereby allowing them to reclaim and reconstitute the meanings of ageist stereotypes in a manner that cushions their impact on the self (Nimrod & Berdychevsky, 2018).

Findings from this study present several implications for research and practice. Although more older adults have been using TikTok since the pandemic (Fishwick, 2020), they nonetheless form only a small fraction of the TikTok user base. The need to consider whether social media environments are age-inclusive is therefore paramount. Evidence suggests that developers of digital technologies are often misled by stereotypes of older adults being technologically inept (Mannheim et al., 2019). Some have also argued that most social media sites are designed by younger cohorts (Marwick, 2013) whose chief aim is to fulfill their peers’ needs, with the needs of older adults added merely as an afterthought (Trentham et al., 2015). While attempts have been made to include older adults in the process of designing social media platforms (Frohlich et al., 2016) and digital technologies in general (LaMonica et al., 2021), older people remain severely under-represented (Rosales & Fernández-Ardèvol, 2020). Work done to address technical concerns has also been effected without due deference to older persons’ opinions (Fischer et al., 2020). Thus, whether the involvement of older individuals in the design process has any material effect in practice remains an open question (Fischer et al., 2020).

Beyond encouraging older persons to use social media, efforts could be made to motivate them to actively create their own content. Not only will this undermine beliefs that older adults are disinterested in technology or passive users of social media (Leist, 2013), it will also embolden them to share their own experiences of later life, thus enabling them to assume a more active role in age-based advocacy. The presence of older people on online venues conventionally monopolized by the young will also give the young exposure to the heterogeneity of the older population, which they may otherwise not encounter in offline settings. Additionally, while some organizations which champion the rights of older persons have developed a presence on social media, most are on Twitter or Facebook. To raise awareness of ageism on a larger scale, these organizations could consider experimenting with short-form content on TikTok where young people predominate. Ultimately, growing old is both an individual and a collective human experience. Given that the internalization of negative age stereotypes may adversely affect one’s health (Levy, 2009), being exposed to positive representations of old age will benefit older individuals who may be struggling to adapt to this new phase of life, and younger people who may have anxiety about growing older.

As research on older TikTokers is still in a fledgling state, directions for future studies are numerous. First, it is plausible that older adults with certain types of personalities are more likely to use TikTok to discuss issues regarding later life. While Omar and Dequan (2020) found that the personality dimensions from the Big Five Personality Model had no significant influence on users’ producing, participating, and consuming behaviors on TikTok, they emphasized that these findings do not foreclose the possibility that personality traits might affect users’ intention to create content. Future studies could expand on this area of research by teasing out the personality traits and motivations that characterize older adults who are more likely to use social media to engage in discourses on old age. Whether their videos are created to influence audiences, for the purposes of monetization, or simply a byproduct of the content they create is a related topic that would benefit from future exploration.

Second, it is important to highlight that while creating content on social media may serve as a creative outlet (Kim, 2012), having a large following online often means having to deal with cyberbullying (Bolat & Gilani, 2018). Although older TikTokers may revel in the opportunity to interact with their younger audiences (Dellatto, 2019), TikTok—not unlike other social media platforms—is not exempt from hateful speech (Han, 2021; Ng & Indran, 2022) or as our study has shown, ageism. A promising avenue for future analysis could therefore shed light on the possible psychosocial benefits and costs that being a public figure on TikTok presents to older adults.

Third, future lines of inquiry could investigate the demographic profile of older adults’ followers as well as the reception of their content by audiences. Since youth occupy the bulk of the TikTok populace, it stands to reason that a sizable segment of these older TikTokers’ followers are younger people previewing possibly their future selves. Some of their followers are also likely to be people from the same age bracket who are making sense of their own identities as aging individuals (McGrath, 2018). Surveys (Ng & Levy, 2018; Ng & Rayner, 2010; Ng et al., 2016; Ng, Allore, et al., 2020), interviews, and big data analytics (Giest & Ng, 2018; Ng, 2018; Ng & Indran, 2021b; Ng & Tan, 2021a; Ng, Lim, et al., 2020; Ng et al., 2021b, 2022a, 2022b) would be instructive in assessing the impact of such content on both younger and older audiences.

Finally, future research could expound on how members of different age cohorts use TikTok to interact with each other. Unlike its rival applications, TikTok is unique in that it has certain features that encourage interaction between users. For instance, the “duet” function enables users to position another user’s preexisting video alongside a new video and is often used by users who wish to “reply” to or “comment” on a particular video (Hutchison, 2020). Likewise, the “stitch” feature allows individuals to reuse snippets of other users’ videos and is frequently used to engage in dialogue (Hutchison, 2020). The newly introduced “Q&A” (Question and Answer) feature allows creators to respond to their followers’ questions in the form of a text or video (Porter, 2021).

This study is not without limitations. First, since we were not privy to the reasons behind the users’ decisions to create the videos, the findings were all subject to our own interpretation. Second, it was beyond the scope of our study to comment on how cultural or demographic factors may have impacted our findings. Since certain societies are more likely to revere old age (Ingersoll-Dayton & Saengtienchai, 1999), cultural values (Ng, 2021b; Ng et al., 2021a, 2021b; Ng & Indran, 2021c; Ng & Lim, 2021; Ng & Tan, 2021b) may have shaped the way in which some of these older TikTokers engaged in discourses on old age. Third, methodological constraints prevented us from analyzing data taken from the “comments” section of the videos. Thus, we were unable to offer insight into how these older TikTok personalities and their followers interact with each other.

## Conclusion

As the older demographic expands in relation to younger groups, their visibility and representation on social media are all the more crucial. Our study reveals the potential for older adults to be put at the vanguard of a movement aimed at challenging socially constructed notions of old age through the use of social media.
